# The emerging role of DEAD/H-box helicases in hepatitis B virus infection

**DOI:** 10.3389/fcimb.2022.1062553

**Published:** 2022-11-25

**Authors:** Hongjuan You, Lihong Ma, Xing Wang, Fulong Zhang, Yiran Han, Jiaqi Yao, Xiucheng Pan, Kuiyang Zheng, Fanyun Kong, Renxian Tang

**Affiliations:** ^1^ Jiangsu Key Laboratory of Immunity and Metabolism, Department of Pathogenic Biology and Immunology, Xuzhou Medical University, Xuzhou, Jiangsu, China; ^2^ Imaging Department, The Second Affiliated Hospital of Shandong First Medical University, Taian, Shandong, China; ^3^ First School of Clinical Medical, Xuzhou Medical University, Xuzhou, Jiangsu, China; ^4^ School of Anesthesiology, Xuzhou Medical University, Xuzhou, Jiangsu, China; ^5^ Department of Infectious Diseases, The Affiliated Hospital of Xuzhou Medical University, Xuzhou, China; ^6^ National Demonstration Center for Experimental Basic Medical Sciences Education, Xuzhou Medical University, Xuzhou, Jiangsu, China

**Keywords:** DEAD/H-box helicases, hepatitis B virus, virus replication, molecular mechanisms, therapy

## Abstract

DEAD/H-box helicases are an essential protein family with a conserved motif containing unique amino acid sequences (Asp-Glu-Ala-Asp/His). Current evidence indicates that DEAD/H-box helicases regulate RNA metabolism and innate immune responses. In recent years, DEAD/H-box helicases have been reported to participate in the development of a variety of diseases, including hepatitis B virus (HBV) infection, which is a significant risk factor for hepatic fibrosis, cirrhosis, and liver cancer. Furthermore, emerging evidence suggests that different DEAD/H-box helicases play vital roles in the regulation of viral replication, based on the interaction of DEAD/H-box helicases with HBV and the modulation of innate signaling pathways mediated by DEAD/H-box helicases. Besides these, HBV can alter the expression and activity of DEAD/H-box helicases to facilitate its biosynthesis. More importantly, current investigation suggests that targeting DEAD/H-box helicases with appropriate compounds is an attractive treatment strategy for the virus infection. In this review, we delineate recent advances in molecular mechanisms relevant to the interplay of DEAD/H-box helicase and HBV and the potential of targeting DEAD/H-box helicase to eliminate HBV infection.

## Introduction

**Figure 1:**
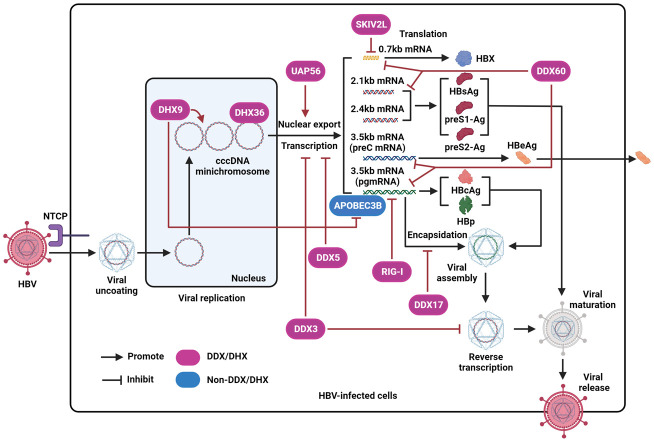
The effect of DEAD/H-box helicases on the regulation of HBV by disrupting different steps of the viral life cycle. After interacting with NTCP and entering host cells, the virus is uncoated and then transferred into the nucleus. Next, the HBV genome is converted into cccDNA, forms a mini-chromosome, and is further transcribed into various viral mRNA, including pregenomic RNA (pgRNA) (3.5kb), preC mRNA (3.5kb), two envelope mRNAs (2.1kb and 2.4kb), and X mRNA (0.7kb). The pgRNA is a translation template for viral polymerase proteins (HBp) and HBcAg. The preC mRNA encodes HBeAg antigen. The two envelope mRNAs encode HBsAg, preS1-Ag, and preS2-Ag. X mRNA codes HBX protein. Then, pgRNA is encapsulated into viral particles and further reverse-transcribed into DNA. Finally, intact viral particles are secreted from liver cells. RIG-I represses HBV pgRNA. DDX60 facilitates the degradation of HBV RNA. DDX3 inhibits the transcription of viral cccDNA and suppresses viral reverse transcription. SKIV2L is capable of degrading HBX mRNA. DDX5 inhibits HBV transcription. DHX9 promotes viral DNA replication, interacts with and inhibits the binding of APOBEC3B to viral pgRNA. DHX36 interacts with the G-quadruplex structure of HBV cccDNA. DDX17 interacts with HBV pgRNA to restrain its encapsidation. UAP56 could facilitate the nuclear export of HBV RNA.

To date, the prevalence of hepatitis B virus (HBV) infection remains high in the Western Pacific region and Africa. HBV is a well-known hepatotropic DNA virus capable of causing persistent infection, which further progresses to hepatic fibrosis, cirrhosis, and liver cancer. To date, the standard therapy for HBV infection has been limited to interferon (IFN), an immunomodulatory agent, and nucleotide analogs, including tenofovir, entecavir, and tenofovir alafenamide, which function as HBV polymerase (HBp) inhibitors. However, these approaches rarely achieve complete viral clearance, and many undesirable adverse effects, including fatigue, headache, and dizziness, often limit the efficacy of the existing antiviral therapies (Tang et al., 2018; Yuen et al., 2018). The HBV genome contains the S, C, X, and P open reading frames (ORFs). S ORF codes HBsAg, preS1-Ag, and preS2-Ag. C ORF contributes to the expression of the structural protein HBcAg and secretory protein HBeAg. X and P ORFs facilitate the production of two regulatory proteins, HBX and HBp. After the virus binds to its receptor sodium taurocholate cotransporting polypeptide (NTCP) and enters liver cells, viral DNA can be transported to the host cell nucleus and converted into closed covalent circular DNA (cccDNA). Depending on a variety of host and viral factors, HBV cccDNA transforms into a minichromosome that acts as a template for viral transcription. Different viral RNAs are then translated into HBV proteins. Sequentially, viral pre-genomic RNA (pgRNA) is encapsulated and reverse-transcribed into HBV DNA. Subsequently, viral DNA-containing particles are enveloped and eventually secreted from the host cells (Fanning et al., 2019; Iannacone and Guidotti, 2022) (**Figure 1**). The interplay between HBV and cellular factors is important for viral replication (Ligat et al., 2021). Hence, targeting host factors that benefit viral infection is a promising strategy for eradicating the virus in HBV-infected individuals.

DExD/H-box helicase is an important RNA-binding protein superfamily of the large super family-2 (SF2) RNA helicases that contributes to the recognition and unwinding of RNA duplexes by specific amino acid motifs in an ATP-dependent manner. Based on the homology of their nucleotide sequences, DExD/H-box helicases are subdivided into DExD-box helicases (DDX) and DExH-box helicases (DHX). A conserved motif with a unique amino acid sequence, D-E-A-D (Asp-Glu-Ala-Asp), exists in DDX and D-E-A-H (Asp-Glu-Ala-His) in DHX (Ullah et al., 2022). Although DHX shares many sequences and structural similarities with DDX proteins, the molecular mechanisms related to RNA regulation, including RNA duplex unwinding, mediated by these two types of DExD/H-box helicases, are different (Gilman et al., 2017). To date, 37 DDX and 16 DHX have been discovered in humans (Andrisani et al., 2022), and accumulating data indicate that they are essential for cellular RNA metabolism, including RNA transcription, RNA splicing, RNA export, microRNA biogenesis, RNA translation, and RNA decay (Ullah et al., 2022). In addition, some DExD/H-box helicases function as DNA sensors and participate in DNA regulation (Hu et al., 2020b; Antcliff et al., 2021). Especially, it has been demonstrated that DExD/H-box helicases play vital roles in multiple biological processes consisting of hematopoiesis, cell proliferation, metabolism, signal transduction, immune response, and inflammation, and are relevant to the development of several diseases, including autoimmune diseases and cancer (Cai et al., 2017; Andrisani et al., 2022; Samir and Kanneganti, 2022). In addition, a variety of DExD/H-box helicases can sense non-self-viral nucleic acids (Fullam and Schroder, 2013; Ullah et al., 2022) and participate in modulating diverse antiviral immune signaling pathways, including Toll-like receptor (TLR) and retinoic acid-inducible gene I (RIG-I)-like receptor (RLR) pathways (Su et al., 2021). Therefore, further advances in understanding the effect of DExD/H-box helicases on viral infection may contribute to the treatment of infectious diseases caused by these viruses.

Recently, many DExD/H-box helicases, including DDX3 (Ko et al., 2014), DDX5 (Sun et al., 2022), DHX9 (Chen et al., 2020), RIG-I (Sato et al., 2015), MDA5 (Lu and Liao, 2013), SKIV2L (DDX13) (Shiromoto et al., 2018), DDX17 (Mao et al., 2021), DHX36 (Meier-Stephenson et al., 2021), and UAP56 (DDX39B), have been identified to play vital roles in the development of chronic HBV infection (Hu et al., 2020a). Multiple molecular mechanisms, including the regulation of HBV replication cycle and the sensitization of the innate immune responses (**Table 1**), are identified to participate in the control of HBV replication and associated liver diseases mediated by these identified DEAD/H-box helicases. Here, we outline the current view of the effect of different DExD/H-box helicases on the modulation of HBV replication, the role of HBV in the alteration of DExD/H-box helicases, and the potential of DExD/H-box helicase-targeting strategies to eliminate HBV infection.

**Table 1:** The detailed information on the interaction between HBV and DEAD/H-box helicases.

Target molecules	The role of target molecules on HBV infection	The biological processes related to HBV mediated by target molecules	The role of HBV on target molecules	The viral protein related to target molecules	The small molecules against target molecules related to the repression of HBV	References
DDX3	Inhibition	HBV life cycle/Innate immune response	Inhibition	HBp	5-HT; AS-19; Rg3	Wang et al., 2009; Yu et al., 2010; Choi et al., 2014; Ko et al., 2014; Kang et al., 2019
DDX5	Inhibition	HBV life cycle/Innate immune response	Inhibition	HBX	Unknown	Zhang et al., 2016; Murphy et al., 2016; Sun et al., 2022
DHX9	Promotion	HBV life cycle	Promotion	HBX	Unknown	Murphy et al., 2016; Shen et al., 2020; Shen et al., 2020
RIG-I	Inhibition	HBV life cycle/Innate immune response	Inhibition	HBX/HBp	poly-U/UC RNA, Poly(I:C)-HMW/LyoVec, Inarigivir	Yu et al., 2010; Jiang and Tang, 2010; [Bibr B49][Bibr B4]; Lee et al., 2021; Fung et al., 2022
MDA5	Inhibition	Innate immune response	Inhibition	HBX	Poly(I:C)-HMW/LyoVec	Wang et al., 2010; Lu and Liao, 2013; Asadi-Asadabad et al., 2021
SKIV2L	Inhibition	HBV life cycle	Promotion	HBX	Unknown	Aly et al., 2016; Shiromoto et al., 2018
DDX17	Inhibition/Promotion	HBV life cycle	Promotion	HBX	Unknown	Mao et al., 2021; Dong et al., 2022
DHX36	Inhibition	HBV life cycle	Unknown	Unknown	Unknown	Meier-Stephenson et al., 2021
DDX60	Inhibition	HBV life cycle	Unknown	Unknown	Unknown	Kouwaki et al., 2016
UAP56	Promotion	HBV life cycle	Unknown	HBX	Unknown	Hu et al., 2020a

## DDX3

DDX3 is a prominent member of the DEAD/H-box helicases involved in the regulation of RNA metabolism and has a pivotal role in antiviral innate immunity (Wang and Ryu, 2010; Ko et al., 2014). Current evidence suggests that DDX3 restricts HBV replication by targeting viral transcription and reverse transcription. For example, by relying on tetracycline-inducible HBV-producing cells, Ko et al. demonstrated that DDX3 inhibited the transcription of viral cccDNA. Although DDX3 interacts with viral transcriptase HBp, DDX3-mediated HBV transcription inhibition is independent of the interplay between HBp and DDX3 (Ko et al., 2014). A common transcription factor, which has not been well identified so far, may plausibly contribute to DDX3-mediated transcriptional suppression of the virus. Wang et al. showed that DDX3 does not affect pgRNA degradation. However, depending on the interaction of HBp with DDX3, DDX3 can be incorporated into nucleocapsids. Furthermore, encapsidated DDX3 had an inhibitory effect on viral reverse transcription. The suppression of HBV reverse transcription mediated by DDX3 may be associated with the disruption of the secondary structure of pgRNA, which is important for HBV biosynthesis in viral nucleocapsids (Wang et al., 2009). More importantly, the mutational analysis indicated that the ATPase activity of DDX3 is vital for the suppression of viral reverse transcription.

**Figure 2:**
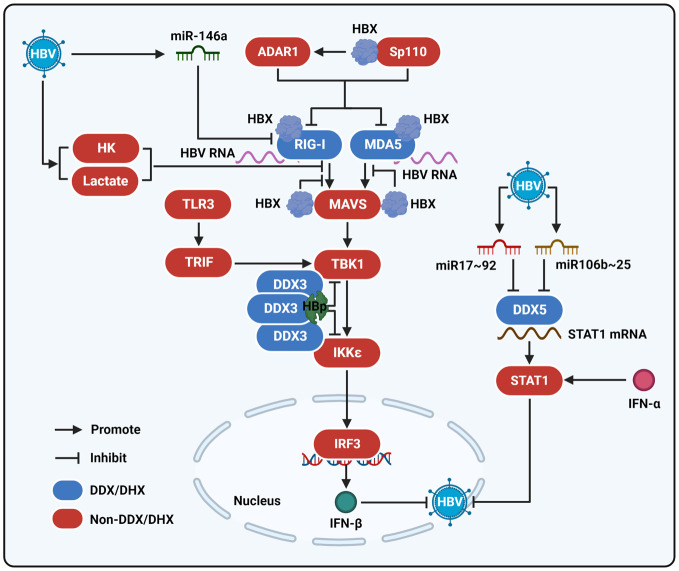
The interaction of HBV with DEAD/H-box helicases to regulate the innate immune response. MDA5 and RIG-I interact with HBV RNA and function as viral RNA sensors to sensitize the RLR signaling pathway. After being stimulated by HBV RNA, RIG-I and MDA5 activate MAVS and then sensitize TBK1. Next, TBK1 stimulates the activation of IKKϵ and IRF3 to produce IFN-β and then repress HBV infection. HBX can interact with MDA5, RIG-I, and MAVS to inhibit their interactions and thereby reduce the activation of the RLR signaling pathway. ADAR1 and Sp110 mediated by HBX also contribute to the modulation of the RIG-I/MDA5-mediated RLR signaling pathway. HBp could interact with DDX3 to suppress the binding of DDX3 to TBK1 or IKKϵ and inhibit the RIG-I/MDA5-mediated RLR signaling pathway, as well as the TLR3 signaling pathway. In addition, HBV can induce miR-164a expression to inhibit RIG-I-mediated innate immune response. HBV upregulates miR106b~25 and miR17~92 clusters to repress DDX5, which binds to STAT1 mRNA and regulates STAT1 translation when the cells are stimulated by IFN-α to suppress viral infection. HBV promotes the activity of hexokinase (HK) and the production of lactate to inhibit the interaction between RIG-I and MAVS.

In addition, DDX3 can strengthen the activity of adapter molecules TANK-binding kinase 1 (TBK1) and IKKϵ, based on its interaction with IKKϵ or TBK1, which further phosphorylates IFN-regulatory factor (IRF) 3 to initiate IFN-β production (Schroder et al., 2008). However, a study by Wang et al. demonstrated that to facilitate HBV infection, HBp can restrain IFN-β production that is triggered by TLR3/TRIF and RIG-I/melanoma differentiation-associated gene 5 (MDA5)-associated RLR signaling pathways that are stimulated by Poly(I:C) in the medium. Poly(I:C) was administered by lipofectin transfection, or treated with Sendai virus, a stimulus of the RIG-I pathway (Wang and Ryu, 2010). Furthermore, pull-down coupled with mass spectrometry (MS) and associated functional experiments revealed that the suppression of TLR3 or RLR signaling pathways mediated by HBp depended on the interaction of HBp with DDX3 to suppress the activity of TBK1/IKKϵ by blocking the binding of DDX3 to IKKϵ to suppress the sensitization of IRF3 and restrain the expression of IFN-β (**Figure 2**) (Yu et al., 2010).

Furthermore, there is evidence that multiple compounds targeting DDX3 have satisfactory effects on the inhibition of HBV infection in cell models. For example, Kang et al. showed the interaction of the serotonin (5-HT) component with its receptor located in hepatocellular cells. This component can increase the DDX3 promoter activity to restrict HBV replication. As previously mentioned, DDX3 can inhibit HBV replication and sensitizes the innate immune response *via* TBK1/IKKϵ/IRF3-mediated IFN-β induction (Wang and Ryu, 2010). A wide variety of 5-HT receptors (from 5-HT1 to 5-HT7 receptors) have been identified, and the agonist of 5-HT7 receptor AS-19 [(2S)-(þ)-5-(1,3,5-trimethylpyrazol-4-yl)-2-(dimethylamino)tetralin] is also known to activate DDX3 and suppress HBV replication by increasing IFN‐β expression (Kang et al., 2019). In addition to AS-19, Choi et al. found that ginsenoside Rg3, an active ingredient in ginseng, exerts anti-HBV activity by elevating DDX3 levels. Mechanistically, the upregulation of DDX3 expression mediated by Rg3 is related to the activation of the DDX3 promoter. Furthermore, p53 phosphorylation mediated by Rg3 contributes to the inhibition of Akt phosphorylation which facilitates DDX3 expression and further activates the TBK1/IKKϵ/IRF3 pathway to inhibit viral replication (Choi et al., 2014).

## DDX5

DDX5 is one of the best-characterized DEAD-box helicases and participates in multiple RNA metabolic processes, including RNA transcription, translation, and decay (Xing et al., 2019). Current evidence indicates that it also plays a vital role in controlling viral replication (Cheng et al., 2018). Zhang et al. showed that DDX5 inhibits viral replication by suppressing the transcription of HBV cccDNA. Detailed investigations have indicated that although DDX5 is an RNA-binding protein, it does not bind HBV RNA. Nevertheless, inhibiting DDX5 can reduce polycomb repressive complex 2 (PRC2) occupancy along with decreased repressive H3K27me3 (Zhang et al., 2016), at the HBV minichromosome formed by viral cccDNA, may leading to increased transcription of HBV pgRNA. Especially, DDX5 could interact with chromatin regulating protein suppressor of zeste 12 (SUZ12), one core subunit of PRC2, and based on the helicase activity, DDX5 enhances the stabilization of SUZ12 by inhibiting ubiquitination-mediated degradation. A recent study demonstrated that SUZ12 has a significant antiviral effect against HBV infection (Wang et al., 2011). Therefore, SUZ12 is speculated to play a crucial role in the inhibition of viral transcription mediated by DDX5.

Additionally, DDX5 enhances the activation of interferon (IFN) signaling to suppress HBV replication. Activation of the JAK/STAT pathway plays a vital role in the antiviral response induced by IFN (Kong et al., 2021b; You et al., 2022). Sun et al. showed that DDX5 could bind to STAT1 mRNA and modulate its translation by resolving a secondary RNA structure, namely the G-quadruplex, which is in the STAT1 mRNA 5’-untranslated region (UTR) to accelerate its expression and activation to facilitate the sensitization of IFN-α signaling (Sun et al., 2022) (**Figure 2**). Conversely, the knockdown of DDX5 *via* small interfering RNA (siRNA) decreased IFN-α-stimulated anti-HBV effects by inhibiting the expression of STAT1.

It has been demonstrated that during HBV replication, DDX5 is downregulated, and reduced DDX5 in HBV-associated hepatocellular carcinoma (HCC) is related to poor prognosis (Mani et al., 2020). In particular, Mani et al. demonstrated that increased miR106b~25 and miR17~92 clusters, including miR-18a, miR-17, miR-19a, miR-20a, miR-19b1, and miR-106b, which bind to the 3’-UTR of DDX5, contribute to the repression of DDX5 induced by HBV (Mani et al., 2020) (**Figure 2**). Furthermore, the decrease in DDX5 induced by HBV activated the Wnt pathway, along with elevated mRNA expression of DVL1, SFRP4, FZD7, SFRP5, and MMP7. In addition, the interaction of DDX5 with lncRNA HOX transcript antisense RNA (HOTAIR) and SUZ12 contributes to the modulation of hepatocarcinogenesis (Zhang et al., 2016). In addition to HBV-associated HCC, the expression of DDX5 declined in the liver tumor tissues of HBX/c-myc bitransgenic mice. In addition, Murphy et al. suggested that DDX5 is a substrate of HBX-DDB1-CUL4-ROC1 (CRL4 HBX) E3 ligase (Murphy et al., 2016). Therefore, it is possible that HBX contributes to DDX5 inhibition during HBV infection, and further investigation is needed to confirm this assumption.

## DHX9

DHX9 also participates in various cellular pathways associated with RNA metabolism and contributes to the regulation of viral infections (Lee and Pelletier, 2016; Guo and Xing, 2021). To date, the expression levels of DHX9 were shown to be increased in HBV-replicating cells and transgenic mice. Shen et al. demonstrated that DHX9 was responsible for viral DNA replication, and the role of DHX9 in HBV biosynthesis relied on its helicase activity and nuclear localization. Furthermore, Nup98, an essential component of the nuclear pore, participates in DHX9-mediated HBV replication (Shen et al., 2020). In addition, HBV can produce circular viral RNAs. DHX9 can bind to HBV circular RNA to modulate the production of viral circular RNA (Sekiba et al., 2018). However, it is still unknown how DHX9 modulates the production of circular viral RNAs during the replication of HBV. APOBEC3B is known to inhibit HBV replication, and its antiviral effect relies on its deaminase activity. To date, the cellular factors that contributed to the anti-HBV effect mediated by APOBEC3B have not been fully defined. Based on co-immunoprecipitation, MS, and associated functional experiments, Chen et al. discovered that the interaction of DHX9 with APOBEC3B has a suppressive effect on the anti-HBV effect of APOBEC3B (Chen et al., 2020). Mechanistically, DHX9 did not affect APOBEC3B deamination activity but inhibited the binding of APOBEC3B to viral pgRNA (**Figure 1**).

The current study indicates that DHX9 upregulation mediated by the virus mainly relies on HBX. Based on tandem affinity purification (TAP)/MS analysis, DHX9 was found to serve as a substrate of the CRL4 HBX E3 ligase complex (Murphy et al., 2016). However, the effect of this E3 ligase complex on DHX9 remains unclear. In addition, Shen et al. suggested that HBX could increase DHX9 protein expression by enhancing its stability. Furthermore, the elevation of DHX9 protein regulated by HBX in hepatocytes is relevant to the inhibition of E3 ligase mouse double minute 2 (MDM2)-associated degradation of DHX9 (Shen et al., 2020).

## RIG-I

RIG-I, also called DDX58, is a well-known immune molecule with the ability to trigger RLR signaling, which is composed of three DExD/H-box RNA helicases: RIG-I, LGP2 (also called DHX58), and MDA5 in the cytoplasm to regulate MAVS activation (Kong et al., 2021b). The structures of RIG-I and MDA5 are similar. They have a helicase domain for RNA sensing, carboxy-terminal repressor domain (CTD) for activity modulation, and caspase activation and recruitment domains (CARDs) for signal transduction. RIG-I mainly binds to short dsRNA of no more than 300 bp, and viral RNA-bearing 5’-diphosphate can activate the RIG-I-mediated IFN response. To date, the molecular nature of MDA5 ligands has not been well identified, but it has been demonstrated that MDA5 is mainly activated by long dsRNAs of over 2 kb pairs. In particular, the presence of specific AU-rich sequences in mRNA, as well as the lack of 2’-O-methylation in mRNA, can be identified by MDA5 (Chan and Jin, 2022). Furthermore, because of their differing preferences for RNA binding, these two molecules can recognize different sections of the same viral genome independently and synergistically (Brisse and Ly, 2019). In HBV infection, whether RIG-1 and MDA5 can recognize HBV RNA in an independent or synergistic manner remains unclear. LGP2 contains only the CTD and helicase domains. Because of the lack of CARDs, LGP2 is considered incompetent and inhibits RIG-I- or MDA5-mediated signaling. After MAVS is activated, it further induces the sensitization of TBK1, which initiates IKK-dependent phosphorylation of NF-κB and leads to IRF3 activation to facilitate the production of inflammatory factors and IFN (You et al., 2022). Current evidence indicates that RIG-I is a viral RNA sensor with the ability to recognize the 5’-ϵ region of HBV pgRNA and induce IFN expression to inhibit infection (**Figure 2**) (Sato et al., 2015). RIG-I counteracts the interplay between HBp and viral pgRNA to restrain viral replication (**Figure 1**). Furthermore, Wu et al. found that RIG-I could increase the IFN-α-mediated immune response by elevating the levels of different antiviral proteins, including PKR, ADAR1, OAS, and Mx (Wu et al., 2018). Knockdown of RIG-I by a specific siRNA also inhibits the phosphorylation of STAT1, a signaling molecule in the IFN-associated immune pathway.

However, to facilitate viral infection, HBV utilizes N6-methyladenosine to repress the RIG-I-mediated recognition of viral RNA (Kim et al., 2020). The virus also blocks the expression of RIG-1 by inducing miR-146a (Hou et al., 2016). Furthermore, HBp and HBX were found to retrain virus-mediated RIG-I-associated signaling. For example, HBp can control RIG-I-induced IFN production by disrupting the interaction of TBK1 with DDX3 (Yu et al., 2010). HBX was found to bind to RIG-I (**Figure 2**) (Jiang and Tang, 2010), and residues Glu119 and Asn118 of the viral protein were mainly responsible for the suppression of RIG-I-related RLR signaling (Wang et al., 2020). HBX also has the ability to interact with MAVS and counteract the interplay between MAVS and RIG-I to block sensitization of the IFN-β promoter (Wang et al., 2010). Additionally, HBX regulates ADAR1 expression to block RIG-I transcription (Wang et al., 2021). Speckled at 110 kDa (Sp110), a transcription factor, can also control the expression of RIG-I (Sengupta et al., 2017; You et al., 2022). The interaction between HBX and Sp110 may downregulate RIG-I. Furthermore, targeting HBX using 5’-triphosphate siRNA can enhance the activation of RIG-I to stimulate the IFN-induced innate response in HBV-infected hepatocytes and pAAV-HBV-transfected mice (Han et al., 2011; Han et al., 2019). In addition, to inhibit RLR signaling, HBV promotes the activity of hexokinase (HK) and the production of lactate, which suppresses the interaction of RIG-I with MAVS and blocks IFN production (Zhou et al., 2021).

Lee et al. evaluated the effect of the RIG-I agonist 5’-triphosphate-poly-U/UC pathogen-associated-molecular-pattern (PAMP) RNA on restraining HBV cccDNA (Lee et al., 2021). The results demonstrated that treating HBV-infected cells with poly-U/UC RNA induces RIG-I signaling activation and causes the upregulation of various antiviral genes, including APOBEC3A, SAMHD1, and APOBEC3G, to repress cccDNA formation and accelerate the decay of HBV cccDNA. In addition, poly (I:C)-HMW/LyoVec, a RIG-1, and MDA5 agonist, was also found to inhibit HBV infection (Asadi-Asadabad et al., 2021), with a decline in HBsAg, HBeAg, and viral cccDNA. Furthermore, the effect of poly (I:C)-HMW/LyoVec was observed to be relevant to the upregulation of APOBEC3.

## MDA5

MDA5 is also known as helicase-DEAD-box protein 116. As mentioned above (You et al., 2022), subsequent to MDA5 sensitization by cytoplasmic dsRNA, the molecule activates the MAVS-associated RLR signaling pathway to cause the production of IFN. A study by Lu et al. indicated that the expression levels of MDA5 were upregulated in HBV plasmid-transfected hepatoma cells and HBV plasmid-injected mouse livers (Lu and Liao, 2013). In particular, the authors revealed that MDA5 associates with HBV-specific nucleic acids, indicating that the molecule can sense HBV to induce the sensitization of RLR signaling, and triggers IFN-β-related innate immune responses with increased expression of MxA and OAS1. To date, the detailed mechanisms that are responsible for the recognition of HBV RNA by MDA5 are not well understood. However, Ebrahim et al. observed that the mRNA levels of MDA5 are significantly attenuated in patients with chronic HBV infection (Ebrahim et al., 2015). Potential explanations for these inconsistencies include differences in the cell lines or clinical specimens used. Among the molecules encoded by the virus, HBX has been observed to suppress MDA5 activation *via* its interaction with MDA5 (Wang et al., 2010) (**Figure 2**). HBX also disrupts the interaction between MDA5 and MAVS to restrain the sensitization of the RLR signaling pathway (Wang et al., 2010). In addition, ADAR1 and Sp110 are linked to a reduction in MDA5 induced by HBX (Sengupta et al., 2017; Wang et al., 2021; You et al., 2022).

## SKIV2L

SKIV2L is required for exosome-mediated RNA surveillance (Lee-Kirsch, 2022). Shiromoto et al. showed that the inflammatory factor IL-1β could upregulate the levels of transcription factor ATF3, which further binds to the cyclic AMP-responsive element sequence in the SKIV2L promoter to benefit its expression. Functionally, SKIV2L binds to HBX mRNA and promotes its degradation to restrict viral infection (Aly et al., 2016; Shiromoto et al., 2018). Mechanistically, with the help of SKIV2L, HBX mRNA can bind to the RNA exosome, and relying on HBS1L-dependent RNA quality control mechanisms, SKIV2L facilitates the decay of HBX mRNA in the RNA exosome (**Figure 1**). Furthermore, the suppression of HBV replication mediated by SKIV2L is IFN-independent. Shiromoto et al. found that HBX significantly increased SKIV2L expression. However, the underlying mechanism remains unknown.

## DDX17

Mao et al. discovered that DDX17 can repress HBV replication in a helicase-dependent manner, by the RNA-binding activity of DDX17, which interacts with the stem-loop structure ϵ of viral pgRNA and then restrains its encapsidation (Mao et al., 2021) (**Figure 1**). However, Dong et al. found that HBX can enhance DDX17 expression (Dong et al., 2022). Upregulation of DDX17 further enhanced viral replication and transcription by increasing ZWINT expression. To date, the effects of DDX17 on HBV replication detected by separate groups have been inconsistent, as mentioned above, and are unknown.

## DHX36

Current evidence shows that, as a member of the DEAD/H-box helicase family, DHX36 can enzymatically unwind G-quadruplex DNA and RNA, which are secondary nucleic acid structures with various roles in different cellular processes (Antcliff et al., 2021). Meier-Stephenson et al. revealed that DHX36 interacts with the G-quadruplex structure in the pre-core promoter region of HBV cccDNA, which is responsible for the generation of viral pgRNA (Meier-Stephenson et al., 2021). The binding of DHX36 to the G-quadruplex of the viral genome may contribute to the regulation of viral replication (**Figure 1**).

## DDX60

DDX60 is an IFN-inducible cytoplasmic DEAD/H-box helicase. Kouwaki et al. found that DDX60 induced by IFN-γ enhanced the degradation of HBV RNA to inhibit HBV infection. Interestingly, the degradation of cytoplasmic viral RNA mediated by DDX60 is faster than that of nuclear viral RNA (Kouwaki et al., 2016) (**Figure 1**). Conversely, the downregulation of DDX60 by siRNA delayed the degradation of cytosolic HBV RNA, but not viral RNA in the nucleus.

## UAP56

UAP56 is a cellular mRNA export factor (Morris et al., 2020). UAP56 benefits HBV replication by binding to HBX to facilitate the nuclear export of viral RNA (**Figure 1**). Furthermore, UAP56 facilitates viral RNA alternative splicing but not transcription. Moreover, the Q-motif in UAP56 is associated with helicase activity and contributes to the interaction between the protein and HBX (Hu et al., 2020a). Downregulation of UAP56 impairs cytosolic accumulation of viral RNA transcripts and reduces the levels of HBV pgRNA splicing variants.

## Conclusion and future perspectives

Here, we show that, depending on various molecular mechanisms, different DEAD/H-box helicases participate in the regulation of HBV replication. On the one hand, some DEAD/H-box helicases, including DDX3, DDX17, DDX5, as well as UAP56, directly participate in the modulation of viral transcription, viral RNA nuclear export, pgRNA encapsidation, and viral reverse transcription in the viral life cycle (**Figure 1**). In contrast, different DEAD/H-box helicases, such as DDX3, MDA5, RIG-I, and DDX5, function as sensors of HBV RNA or modulate the function of adapter molecules or the translation of distinct genes in the RLR, TLR3, and IFN-related antiviral signaling pathways to trigger the innate immune response (**Figure 2**). In addition, inhibition of HBV replication mediated by DDX41 and DHX35 has been reported (Aly et al., 2016). Nevertheless, the underlying mechanisms are still unclear. It should be noted that, although DEAD/H-box helicases are involved in HBV infection, the effect of the identified DEAD/H-box helicases on viral replication varies. Among the identified DEAD/H-box helicases, only DHX9 and UAP56 have been identified to benefit HBV replication, while other DEAD/H-box helicases exhibit an anti-HBV effect. As stated before, 53 DEAD/H-box helicases have been identified to date (Andrisani et al., 2022). However, only a few DEAD/H-box helicases, presented in this review, have been found to modulate HBV replication. In the future, more research is worthy to elucidate the interplay between DEAD/H-box helicases and HBV.

The current standard therapy for HBV is based on IFN and nucleus(t)ide analogs (Tang et al., 2018; Fanning et al., 2019). IFN treatment elicits the antiviral immune response. Nucleus(t)ide analogs restrict viral biosynthesis by disrupting HBp activity. However, these treatments cannot eliminate the virus, and the main treatment goal is to prevent disease progression and improve survival and quality of life. Moreover, current approaches often result in adverse side effects, therapeutic resistance, and recurrence of the disease (Feng et al., 2018). Therefore, new molecular targets are urgently required to improve the therapeutic effects. Because of the potential significance of DEAD/H-box helicases in the modulation of viral replication by targeting different steps in the HBV life cycle or participating in antiviral immune signaling pathways, targeting these molecules is an attractive strategy to attenuate HBV infection.

More importantly, our review indicates that the use of different small molecules to target DDX3 and RIG-I can effectively repress HBV replication (Choi et al., 2014; Kang et al., 2019; Lee et al., 2021). In particular, the RIG-I agonist, inarigivir, has undergone clinical trials for the treatment of HBV infection in a small study. It has been demonstrated that inarigivir can cause HBsAg reduction of more than 1 log10 IU/ml in 55% of patients. Nevertheless, 17% of patients had significant ALT flares. One patient with necrotizing pancreatitis was dead, and drug-induced liver steatosis and injury were also observed. Owing to these serious adverse reactions, clinical trials of inarigivir have been terminated (Fung et al., 2022). In addition to inarigivir, the antiviral effects of other RIG-1 agonists, including a synthetic 5’-triphosphate dsRNA RIG-I ligand (3pRNA), stem-loop RNA 14 (SLR14), and a sequence-optimized RIG-I agonist (named M8), have been explored by different groups (Chiang et al., 2015; Mao et al., 2022; Marx et al., 2022). In the future, the role of these RIG-1 agonists in the treatment of HBV infections should be examined. However, compounds targeting other DEAD/H-box helicases, including DDX5, DHX36, and UAP56, have not yet been discovered. In recent years, many significant breakthroughs have been achieved in the development of DEAD/H-box helicase inhibitors to treat cancer, and the efficacy of some DEAD/H-box helicase inhibitors has been examined in preclinical studies (Cai et al., 2017). To better assess whether targeting DEAD/H-box helicase is an effective therapeutic strategy against HBV, more attention is needed to develop compounds targeting DEAD/H-box helicases and explore their effect on the treatment of HBV infection.

During HBV infection, viral proteins including HBX and HBp have evolved multiple strategies to regulate the expression of different DEAD/H-box helicases to facilitate infection. Especially, HBX is a multifunctional viral molecule that is essential for HBV replication and associated diseases (Kong et al., 2019; Kong et al., 2021a; Kong et al., 2021c; You et al., 2022). This review examines various evidence that indicates HBX regulates different DEAD/H-box helicases, including DHX9, RIG-I, and MDA5. However, the underlying mechanisms related to HBX-induced modulation of DEAD/H-box helicases remain poorly understood, and further investigation is required. In addition, recent studies have mainly focused on the effects of DEAD/H-box helicases on HBV replication. In addition, DDX3 (Chang et al., 2006), DDX5 (Mani et al., 2020), DHX15 (Xie et al., 2019), and DDX17 (Dong et al., 2022), are associated with HBV-associated HCC. The role of DEAD/H-box helicases in the progression of different liver diseases, including hepatic fibrosis, cirrhosis, and HCC, caused by HBV infection, is poorly understood. Hence, it is vital to assess the effects of different DEAD/H-box helicases, and the relevant molecular mechanisms involved in the modulation of diverse diseases induced by the viruses in the future.

## Author contributions

HY and LM contributed the work equally. HY, LM, XW, FZ, YH, JY, and XP designed the artwork and wrote the manuscript. KZ, FK, and RT supervised the manuscript. All authors contributed to the article and approved the submitted version.

## Funding

The study was supported by the Natural Science Foundation of Jiangsu Province (BK20211347), the Natural Science Foundation of the Jiangsu Higher Education Institutions (21KJA310004), Xuzhou Technology Bureau Foundation (KC21065), and a project funded by the Priority Academic Program Development of Jiangsu Higher Education Institutions (PAPD). The Figures used in the review was created by BioRender (https://biorender.com/).

## Conflict of interest

The authors declare that the research was conducted in the absence of any commercial or financial relationships that could be construed as a potential conflict of interest.

## Publisher’s note

All claims expressed in this article are solely those of the authors and do not necessarily represent those of their affiliated organizations, or those of the publisher, the editors and the reviewers. Any product that may be evaluated in this article, or claim that may be made by its manufacturer, is not guaranteed or endorsed by the publisher.
